# A Numerical Study on Lightning Damages and Residual Strength of CFRP Laminates Considering Delamination Induced by Thermal Stress

**DOI:** 10.3390/polym17162245

**Published:** 2025-08-19

**Authors:** Qian-Zhi Yin, Jiapeng Bian, Yin Fan

**Affiliations:** School of Aeronautics and Astronautics, Shanghai Jiao Tong University, Shanghai 200240, China; yqz0402@sjtu.edu.cn (Q.-Z.Y.); jiapengbian@sjtu.edu.cn (J.B.)

**Keywords:** CFRP laminate, lightning strike damage, delamination, cohesive zone method, residual compressive strength

## Abstract

Most numerical studies on carbon fiber-reinforced polymer (CFRP) lightning damages fail to account for delamination, a factor that plays a significant role in the subsequent analysis of residual strength. This study establishes an electro-thermo-mechanical coupled numerical model incorporating delamination effects to predict lightning-induced damage in carbon fiber-reinforced plastic (CFRP) composites. Subsequently, parametric investigations evaluate the influence of varying input loads and stacking sequences on interlaminar pyrolysis and delamination damage, with damage assessment quantitatively conducted based on simulated post-strike uniaxial ultimate compressive loads. Post-strike uniaxial compressive strength reduction with cohesive elements is 28.91%, demonstrating closer alignment with experimental reduction (36.72%) than the 21.12% reduction predicted by the interlaminar-effect-neglecting model. Under combined thermal expansion and shockwave overpressure, the 28.91% compressive strength reduction demonstrates closer alignment with the experimental 36.72% reduction than the 25.13% reduction observed under isolated shockwave overpressure. The results highlight the critical role of thermal delamination in compressive strength reduction, with distinct waveform-dependent mechanisms: under C-waveform lightning currents, arc thermal effects cannot be neglected; D-waveform strikes exhibit predominant contributions from impact loading to delamination damage, with thermally driven delamination likewise pronounced. Increased current amplitude correlates with amplified mechanical damage severity, while premature symmetry in ply stacking sequences exacerbates compressive performance degradation. This work enhances multi-physics modeling fidelity by bridging thermal delamination and mechanical degradation pathways, offering foundational insights for optimizing lightning strike resistance in advanced aerospace composite systems.

## 1. Introduction

As reported, commercial aircraft typically experience approximately one natural lightning strike annually, which severely threatens flight safety, especially for those with large composite structures [1,2,3,4]. When subjected to a lightning strike, the composite laminates always demonstrate complex damage modes due to their inherently complex internal structure. While distinct composite materials exhibit varying performance advantages in alkaline and aqueous immersion environments [5], CFRP demonstrates superior mechanical properties relative to GFRP and alternative composites under standard operational conditions, specifically non-extreme environments characteristic of airworthy flight regimes [6]. This established performance advantage positions CFRP as the prevailing choice for aerospace applications. When subjected to lightning strikes, a composite laminate experiences a significant temperature rise, degrading its structural integrity. As temperatures approach the resin’s glass transition temperature, interfacial bonding between fibers and resin weakens. Further heating initiates resin pyrolysis, increasing internal porosity and progressively degrading interfacial adhesion, ultimately impairing fiber load-sharing capability. Under sustained high temperatures, oxidative degradation of exposed carbon fibers reduces fiber diameter, further reducing overall load-bearing capacity [7]. Lightning-induced damage in carbon fiber-reinforced plastic laminates manifests primarily in three distinct modes: fiber fracture, resin decomposition, and internal delamination [8]. Assessing the extent of lightning strike damage in composite laminates remains crucial for aircraft design, maintenance, and inspection.

Lightning strike damage behavior in CFRP laminates has been preliminarily assessed through simulated lightning tests [9,10,11,12,13,14,15,16,17]. Experimental results demonstrated that under high-current conditions, electrical currents traversing CFRP generate significant Joule heating, causing rapid temperature rise. As temperature increases, the CFRP matrix undergoes pyrolysis and gasification, leading to internal bursting. When temperatures reach the sublimation point of carbon, carbon fibers sublimate. When exposed to lightning strikes, composite laminates undergo rapid temperature escalation. This transient thermal loading initiates resin pyrolysis and fiber oxidation, potentially culminating in fiber sublimation. Concurrently, non-uniform heating induces differential thermal expansion, while gas generation may trigger explosive rupture, collectively contributing to mechanical damage. Post-strike evaluations via non-destructive techniques (including SEM, ultrasonic C-scan, and CT scanning) and uniaxial mechanical testing reveal substantial matrix damage, fiber breakage, and interlayer delamination in CFRP, accompanied by significant compressive strength degradation [9,10,11,12,13,14,15,16,17]. However, a comprehensive understanding of the underlying damage mechanisms requires both experimental and numerical methods [18,19,20,21,22,23,24,25,26,27,28,29,30,31]. Ogasawara et al. [18] performed coupled electro-thermal simulations to analyze thermal damage in CFRP laminates with temperature-dependent electrical properties under single-pulse waveforms, with calculated high-temperature zones substantially aligned with Hirano’s experimental damage regions [8]. Dong et al. [19] enhanced the model’s validity by incorporating temperature- and time- dependent parameters, modeling electrical and thermal properties as functions of resin pyrolysis degree. However, these studies primarily addressed thermal ablation damage. Crucially, CFRP laminates experience not only thermal damage (matrix pyrolysis, fiber sublimation) but also structural damage from impact loading, Lorentz forces, and thermal stresses during lightning attachment [20,21], making pure thermal simulations insufficient for predicting residual load-bearing capacity. Recent electro-thermo-mechanical methodologies show promise: Karch et al. [22] computed elastic response and intralaminar damage in protected CFRP structures (accounting for lightning protection layer explosions and arc-root behavior), which yielded improved test correlation while neglecting interlaminar effects; Dong et al. [23] established a stiffness degradation model using Continuum Damage Mechanics (CDM) to simulate the effect of thermal ablation and expansion on CFRP, revealing synergistic thermal–mechanical damage origins; and Bian et al. [24] predicted residual compressive strength of a CFRP laminate subjected to a lightning strike using Hashin’s criterion on extended damage zones, quantitatively assessing mechanical degradation resulting from lightning strike damages. Moreover, Dong et al. [25] proposed a data-driven prediction for the residual strength of CFRP laminates with lightning damage, opening a novel avenue for analyzing the effect of lightning strike damage on mechanical properties of CFRP.

In fact, when integrating the degradation of mechanical properties in the systematic assessment of the effects of lightning strikes on CFRP, delamination damage—a typical failure of laminates arising from interlaminar stress concentrations [26]—is crucial, especially for accurately predicting residual compressive strength [27].

Albertino et al. [28] computed intralaminar and interlaminar lightning-induced damage alongside mechanical responses in protected CFRP structures, implementing progressive damage analysis to simulate interlaminar damage under impact loading for assessing lightning strike effects on laminate performance. While their research concurrently evaluated both intralaminar and interlaminar mechanical damage induced by lightning strike-induced shockwaves, it did not incorporate the influence of material pyrolysis on mechanical properties at intra-/interlayer scales. Consequently, Naghipour et al. [29] designed multidirectional composites incorporating user-defined, temperature-dependent cohesive elements, to investigate delamination propagation under specified conditions. Their study revealed a strong dependency of delamination zone geometry on assumed temperature-dependent fracture toughness, providing insights into impact-driven delamination via temperature-sensitive cohesive zone modeling (CZM) while omitting thermally driven delamination due to thermal expansion. Yang et al. [30] employed sequential thermo-mechanical FE coupling with dielectric breakdown modeling to simulate lightning damage in woven CFRP (W-CFRP) laminates and woven composite honeycomb sandwich panels (W-CHSPs), applying 200 kA surface currents along with corresponding lightning surge overpressures. Using FE models integrated with LaRC05 criteria, they characterized intralaminar damage and interlaminar delamination in both systems, accounting for impact-induced degradation of thermally compromised mechanical properties—yet neglecting thermal expansion effects and omitting quantitative degradation analysis. Notably, mismatches between in-plane and through-thickness coefficients of thermal expansion (CTEs) generate interlaminar normal and shear stresses when laminates experience constrained thermal deformation, inducing thermal delamination. Complementarily, Kamiyama et al. [31] systematically evaluated pyrolysis behavior via thermogravimetric analysis, sequentially performing coupled electro-thermal simulations, heat transfer analysis, and thermal delamination assessment to investigate interlaminar thermal decomposition in CFRP laminates under lightning hyperthermal environments. Their work quantified the extent of pyrolysis area but neither analyzed thermal delamination damage quantitatively nor considered the effects of impact loading on delamination propagation.

Recent advancements in numerical modeling for lightning-struck CFRP laminates have progressively addressed intralaminar pyrolysis, interlaminar adhesive thermal debonding, shockwave-induced intralaminar damage, and delamination, along with preliminary investigations into thermal expansion-mediated delamination. Despite these developments, the existing frameworks remain incomplete in characterizing mechanical performance degradation arising from thermally driven delamination during lightning events. For instance, Dong’s work [23] focused solely on intralayer thermal expansion damage while neglecting interlaminar effects; although Naghipour [29] and Yang [30] et al. introduced interfacial cohesive elements, they considered only impact-induced delamination without accounting for delamination induced by thermal expansion during lightning strikes. The limited scope of their models resulted in underestimated damage predictions in simulations. Concurrently, existing studies on lightning damage modeling of laminated composites with cohesive elements primarily emphasize delamination area, with insufficient exploration of post-lightning mechanical property degradation. To address these gaps, this study establishes a comprehensive lightning damage model by integrating thermal expansion, mechanical impact loading, and interfacial cohesive elements within a unified framework. The model specifically addresses thermal expansion processes during lightning events and associated delamination mechanisms arising from mismatched thermal expansion across plies, implementing an integrated simulation workflow encompassing full lightning discharge, heat dissipation, and post-strike compression analysis. Numerical simulations of compressive performance pre-lightning and post-lightning exposure were implemented and validated via standardized lightning strike testing protocols and mechanical characterization, establishing a predictive residual strength framework that explicitly integrates DTS (delamination induced by thermal stress) effects. Parametric studies further examined the influence of lightning waveform characteristics (C-wave versus D-wave), current amplitude variations, and ply orientation configurations on damage progression.

## 2. Models and Methods

To systematically investigate the thermodynamic damage evolution in composite laminates during lightning strike events, this study employs a decoupled simulation approach that sequentially addresses the lightning current injection phase and subsequent cooling process through coordinated electro-thermal–mechanical modeling. The methodology first applies an electro-thermal coupling model to simulate the transient thermal ablation phenomena during lightning current injection, establishing the fundamental temperature distribution and pyrolysis degree field within the laminate. Building upon these thermal analysis results, a sequential thermo-mechanical coupling framework is then implemented to characterize the thermal expansion behavior induced by material degradation, effectively capturing the interdependent thermal–mechanical responses during the active strike phase. During the post-strike cooling simulation phase, the progressive thermal damage propagation is tracked through continued thermo-mechanical coupling analysis, while specifically evaluating the thermal expansion damage mechanisms associated with pyrolyzed interlayer adhesives. This integrated modeling strategy not only eliminates the confounding effects of residual thermal stresses in subsequent uniaxial compression simulations but also isolates and quantifies the critical influence of thermally induced delamination on the structural integrity and compressive performance degradation of lightning-damaged laminates.

### 2.1. Material Properties

**Figure 1:**
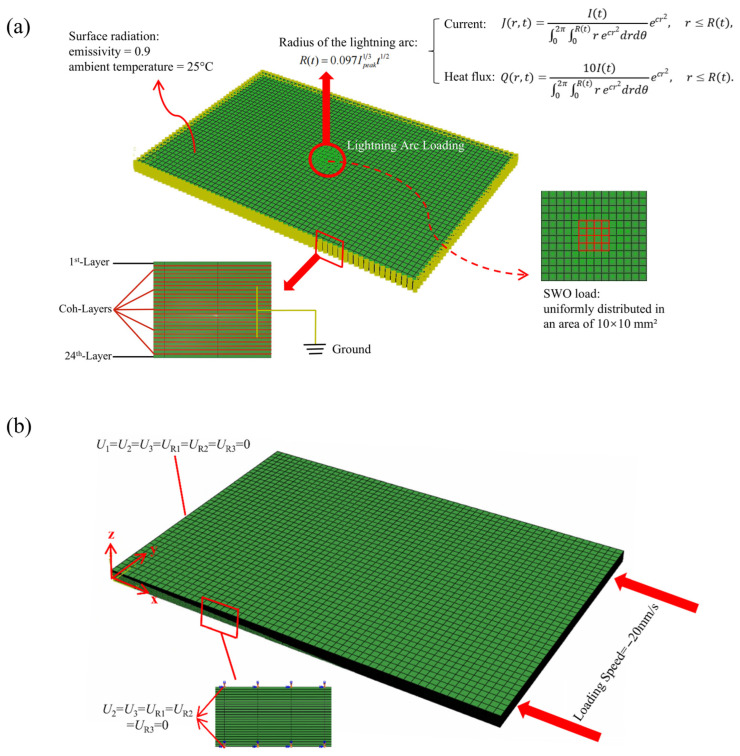
The finite element model: (**a**) lightning strike process; (**b**) compression process after impact.

Figure 1 presents a finite element model of a specimen subjected to an electrical current impulse. In the figure, the 1st-Layer denotes the laminate ply that is first subjected to lightning strikes, the Coh-Layers represent the cohesive interlayers, *R*(*t*) is the expansion radius of the arc with time, *J*(*r,t*) represents the surface current distribution, and *Q*(*r,t*) represents the surface heat flux distribution. The mathematical formulations characterizing the arc channel radius evolution, current density, and heat flux density were established by Wang and Zhupanska [32,33]. Each lamina of the composite laminate was divided into an eight-node isoperimetric solid element in the thickness direction (0.187 mm × 24 plies). The thin solid elements (0.04 mm) adopted at respective interlayers were used as cohesive elements for stress analysis (delamination analysis), as explained later. The thermo-electric properties of the materials are shown in Table 1 [34]. A lightning waveform based on simulated lightning current testing was applied to the center of a specimen surface. The side and bottom surfaces of the specimen were grounded through a copper jig. The electrical potential is regarded as zero. Thermal radiation is given for the specimen surfaces. The emissivity and environmental temperature were, respectively, 0.9 and 25 °C. When the temperature exceeds 3000 °C, the sublimation rate of carbon increases exponentially, suggesting that the Joule heat generation is terminated due to carbon fiber breaks. Therefore, the upper limit of the temperature used for analysis was set as 3000 °C by the application of virtual latent heat.

A unidirectional composite lamina can be modeled as an orthotropic material. In this framework, the damage-integrated stiffness matrix ***C****_d_* for the CFRP material is explicitly defined as follows:

(1) $$C_{d}=\left[\begin{array}{cc} \begin{array}{ccc} df\times C_{11} & df\times dm\times C_{12} & df\times dz\times C_{13}\\ df\times dm\times C_{21} & dm\times C_{22} & dm\times dz\times C_{23}\\ df\times dz\times C_{31} & dm\times dz\times C_{32} & dz\times C_{33} \end{array} & \\ & \begin{array}{ccc} df\times dm\times C_{44} & & \\ & df\times dz\times C_{55} & \\ & & dm\times dz\times C_{66} \end{array} \end{array}\right]$$

where *df* represents the damage variable along the fiber direction, while *dm* and *dz* denote the damage variables associated with transverse and through-thickness directions. These variables have a value of 1 when the material is intact. They degrade gradually to 0 upon material failure.

During the lightning strike process, when the temperature reaches a certain range, the resin will undergo pyrolysis, at which point the mechanical properties of CFRP will degrade. The degree of pyrolysis can be expressed by

(2) $$\frac{d\alpha }{dT}=\frac{A}{\beta }{\left(1-\alpha \right)}^{n}\exp(-\frac{E_{a}}{RT})$$

The relationship of the degree of thermal cracking is as follows:

(3) $$\alpha (T)=\left\{\begin{array}{l} 0,\;\;T\leq 523\;K\;\\ 0.46+0.57\times [\coth(\frac{T-642.88}{23.29})-\frac{23.29}{T-642.88}],\;\;523\;K<\;T\;<\;873\;K\\ 1,\;\;T\geq 873\;K \end{array}\right.$$

The stiffness matrix of material incorporating thermal damage is expressed as $C_{d}(T)=[1-\alpha (T)]C_{d}$. It is stipulated that when the maximum temperature of the unit is less than or equal to 523 K, the material has no ablation damage; when the unit temperature exceeds 523 K, the matrix undergoes ablation, defined as ordinary ablation damage; and when the unit temperature exceeds 873 K, both the fiber and matrix experience ablation damage, defined as severe ablation damage [35].

When analyzing the failure of CFRP with lightning strike damage, a strain-based 3DHashin failure criterion is used for predicting the residual compressive strength of CFRP laminates. The 3DHashin criterion classifies potential failure modes into six distinct categories based on the strain state of the element: fiber tension, fiber compression, matrix tension, matrix compression, through-thickness tension, and through-thickness compression. Following identification of the applicable failure mode through strain-state evaluation, the corresponding failure criterion is computed. Damage initiation is deemed to occur when this dimensionless index exceeds unity. The ambient-temperature mechanical properties of the laminate are summarized in Table 2.

**Figure 2:**
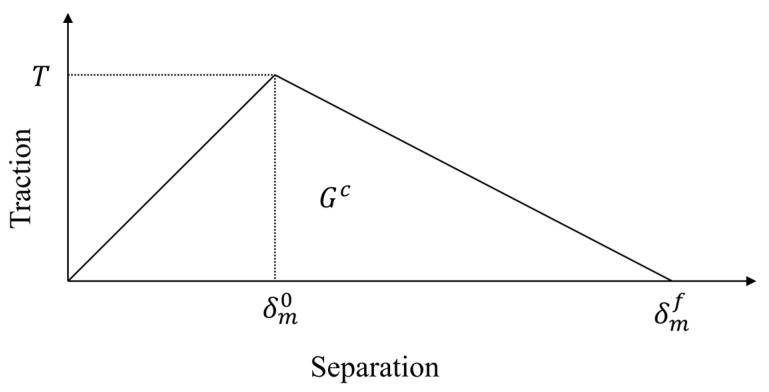
CZM traction–separation curve.

To characterize the mechanical failure of interlaminar discontinuous interfaces, the cohesive zone model was implemented to predict delamination damage progression. The cohesive zone model characterizes delamination damage through predefined cohesive elements at interlaminar interfaces governed by a traction–separation law. Damage initiates when interfacial stress reaches the strength threshold, followed by progressive stiffness degradation with increasing separation displacement. Final failure occurs upon reaching the critical displacement, resulting in complete interfacial decohesion. The relation between the separation and traction of cohesive elements is shown in Figure 2. The energy dissipation within the element causes crack propagation. Regarding delamination progression under a mixed mode, the power law of the energy release rate and the critical energy release rate of each mode was applied [37,38]. The temperature of each cohesive element in every increment was taken from the coupled thermal–electrical analysis and heat transfer analysis results described in the previous section, because thin interlayer solid elements were applied to correspond to the cohesive elements. The interlaminar properties are summarized in Table 3.

### 2.2. Lightning Strike Process

Figure 1a presents the finite element model of the lightning strike process. The simulation of thermal ablation induced by current injection is conducted using a thermo-electrical coupling model. When electrical current traverses the laminate, the internal electric field distribution can be described by Maxwell’s equations. According to the charge conservation equation, the finite element form of the electric field control equation is as follows:

(4) $$\int_{V}\frac{\partial \delta \varphi }{\partial x}\cdot \sigma^{E}\cdot \frac{\partial \varphi }{\partial x}dV=\int_{S}\delta \varphi JdS+\int_{V}\delta \varphi \tau_{c}dV$$

In this formulation, ***J*** denotes the current density vector, *S* is the surface area, *τ_c_* signifies the current source per unit volume, *V* corresponds to the differential volume element, *E* is the electric field intensity, *φ* is the electric potential, and ***σ****^E^* represents the temperature-dependent electrical conductivity matrix. The conductivity changes with temperature.

The internal electrical circuit of the laminate is the internal heat source for Joule heating. According to the thermal energy balance equation, the micro-element heat conduction control equation is

(5) $$\int_{V}\rho C_{V}\frac{\partial \theta }{\partial t}\partial \theta dV+\int_{V}\nabla \partial \theta \cdot \left(K\cdot \nabla \theta \right)dV=\int_{V}\delta \theta rdV+\int_{S}\delta \theta qdS$$

In the governing equations, *θ* denotes temperature, *K* represents the thermal conductivity matrix, *ρ* signifies material density, *C_V_* corresponds to specific heat capacity, and *q* indicates heat flux density per unit area.

The temperature field distribution obtained from the thermo-electric coupling simulation during lightning strikes is used as a heat source input for the thermal expansion simulation calculations during the lightning strike process. In the thermal expansion process, the edges of the laminated plate are fixed to simulate the lightning strike test fixture, and the mechanical properties of the materials are simultaneously examined as they degrade with thermal decomposition. The model applies CZM to calculate the interlaminar mechanical relationships in order to simulate and compute the delamination damage caused by thermal expansion. In D-wave simulations, the lightning strike-induced shockwave overpressure (SWO) constitutes a significant factor. The calculated SWO magnitude corresponding to the D-wave lightning current with a 40 kA peak amplitude reaches 19.47 MPa [41].

Current injection experiments were conducted on CFRP laminates fabricated from M21C prepreg through hot-press curing. The current application protocol employed a central copper wire electrode for controlled arc injection on the upper surface, with conductive copper foil electrodes attached to side surfaces to maintain zero potential grounding. The input waveforms conformed to the SAE Standard [42], comprising Waveform D (40 kA peak amplitude) and Waveform C (200 A peak amplitude), to simulate standardized lightning strike conditions.

### 2.3. Heat Dissipation Process

During the cooling process to room temperature, the convection heat transfer boundary conditions cannot be ignored due to the longer cooling time compared to the lightning strike duration. The four sides of the cooling process are kept fixed, and all surfaces except the upper surface are adiabatic. When simulating the cooling process, a subroutine needs to be used to record the highest temperature at each grid node during the historical time increment steps, in order to update the thermal degradation of the grid in real time using the highest temperature, thereby representing the irreversible process of thermal degradation.

### 2.4. Compression Process After Lightning Strike

Figure 1b presents the finite element model of the compression process after a lightning strike. Uniaxial compression is applied in the *x*-direction. During the loading process, the boundary conditions are set according to the ASTM standard [43], the *x* = 0 section is fully fixed, and the four edges of *y* = *z* = 0 mm, *y* = 0 mm and *z* = 5.488 mm, *y* = 50 mm and *z* = 0 mm, and *y* = 50 mm and *z* = 5.488 mm constrain all five spatial degrees of freedom except for translation along the *x*-axis. The loading mode is displacement loading, and the loading speed is 20 mm/s.

## 3. Results and Discussions

**Figure 3:**
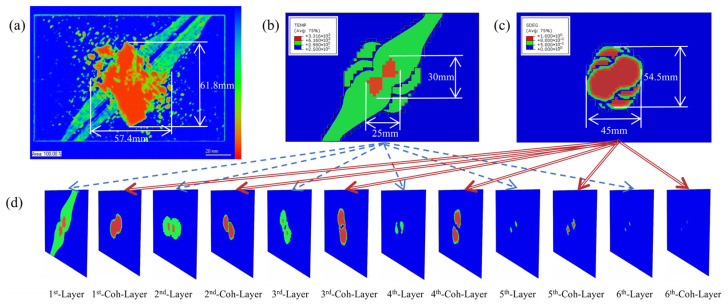
Thermal damage contour plot of laminated plate subjected to D-wave current: (**a**) Ultrasonic C-scan contour plot of simulated lightning strike test. (**b**) Laminate thermo-electric ablation damage cloud diagram of finite element model with cohesive layer. Blue areas indicate no ablation damage, green areas represent partial matrix ablation, and orange areas show complete matrix pyrolysis. (**c**) Interlayer thermal damage model of finite element model with cohesive layer. Blue areas indicate undamaged regions, green areas represent pyrolyzed zones, and orange areas denote thermal delamination regions. (**d**) Damage contours across multiple layers.

Figure 3 illustrates the thermal damage in the [45/0/−45/90]_3s_ laminate subjected to a 40 kA Component D lightning current waveform. Figure 3a–c present the experimental ultrasonic C-scan image, the simulated intralayer thermo-electric ablation damage distribution (including cohesive delamination), and the simulated interlayer DTS contours, respectively.

In the ultrasonic C-scan image, the intensity of echo signals received by the probe is depicted using a color map: warmer hues indicate lower decibel levels of reflected waves and greater ultrasonic energy loss. In the CFRP lightning strike test specimen, the central orange region exhibits severe ultrasonic attenuation. Combined with CFRP lightning damage mechanism analysis, this area demonstrates significant ablation and severe delamination damage. Green regions along the 45° direction show partial signal attenuation, where delamination is less pronounced. The ultrasonic attenuation in these regions primarily results from energy absorption by intracavity voids formed due to resin pyrolysis along fiber directions in the surface layer. As the model does not account for internal gas explosion effects, the simulated delamination area is smaller than that measured by ultrasonic C-scan.

**Figure 4:**
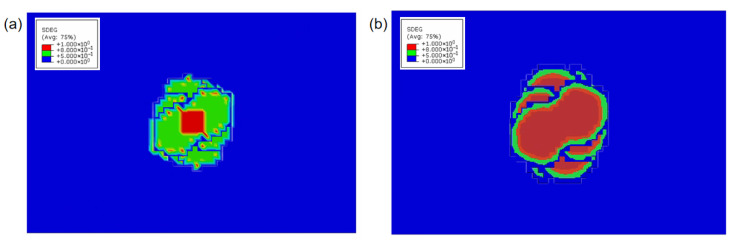
Thermal damage contour plot of the composite laminate subjected to D-wave current: (**a**) damage contours that consider only impact damage; (**b**) damage contours that account for impact damage and thermal stratification.

Figure 4a displays the delamination damage contour resulting from a uniformly distributed 19.47 MPa impact load applied solely to the central 10 mm × 10 mm region of the upper surface. Figure 4b presents the delamination contour incorporating both impact loading and thermally induced stresses. Contour analysis reveals that under D-wave conditions, pyrolytic degradation of cohesive elements significantly influences delamination damage computation through fracture toughness reduction, while thermally driven delamination damage due to thermal expansion remains non-negligible. Under isolated shockwave overpressure, the residual strength is 192.79 kN, representing a 25.13% reduction from the undamaged compressive strength. With combined thermal expansion and shockwave overpressure, the residual strength measures 183.06 kN, corresponding to a 28.91% strength reduction. This combined-effect reduction (28.91%) demonstrates closer alignment with the experimental strength reduction (36.72%) than the isolated overpressure scenario (25.13%).

**Figure 5:**
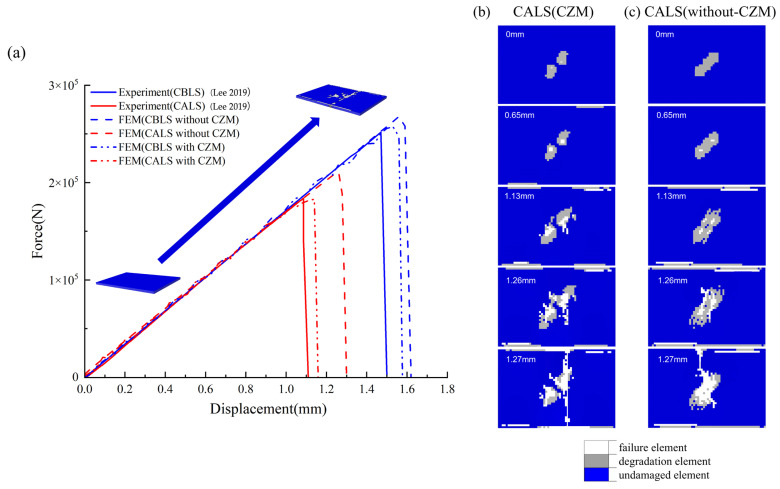
(**a**) Single-axis compression load–displacement curve [21]; (**b**) illustration of damage propagation on the top surface of the laminated plate during uniaxial compression after a lightning strike (with CZM); (**c**) illustration of damage propagation on the top surface of the laminated plate during uniaxial compression after a lightning strike (without CZM).

To quantitatively evaluate the influence of thermal expansion-induced delamination on mechanical performance degradation, quasi-static uniaxial compression simulations along the longitudinal edge were conducted. The simulation results from the thermal delamination model incorporating pyrolyzable interlaminar adhesive layer elements were compared with experimental data and contrasted against simulations neglecting thermal delamination effects. The load–displacement curves, as shown in Figure 5a, demonstrate that the thermal delamination model with cohesive elements exhibits closer alignment with experimental ultimate compressive loads compared to models solely considering intralaminar mechanical degradation. Analysis of the load–displacement curves reveals that introducing cohesive elements reduces the simulated ultimate compressive strength of initially damage-free laminates. In these defect-free models, no delamination occurs prior to peak load attainment. However, upon structural failure initiation, significant delamination develops concurrently with transverse matrix cracking. The inclusion of shear-sensitive cohesive elements thus causes slight degradation in overall compressive resistance. Conversely, when modeling laminates containing initial lightning-induced damage, cohesive elements substantially reduce the finite element model’s compressive load-bearing capacity. Figure 5b,c illustrate damage progression on the lightning-struck top surface of the laminated plate during uniaxial compression, with displacement-dependent evolution. Figure 5b shows simulation results incorporating cohesive elements, while Figure 5c corresponds to the model neglecting interlaminar effects. Contour analysis reveals that during initial compression, the cohesive-element model exhibits a smaller damage area than the non-cohesive model. This reduction is attributed to energy absorption through delamination, which mitigates intralaminar damage. However, as displacement increases, the element deletion region expands significantly faster in the delamination-enabled model. This model reaches ultimate compressive strength at 1.13 mm displacement, where transverse matrix cracks initiate on the top surface. By contrast, the non-cohesive model shows no fracture initiation at this stage, reaching its load–displacement curve inflection point only at 1.26 mm displacement. During post-lightning uniaxial compression simulations, finite element models with initial delamination defects reach structural failure earlier than models neglecting interlaminar effects. Although the initial delamination damage exhibits no propagation prior to failure, its presence accelerates intralaminar damage initiation, thereby promoting earlier onset of in-plane matrix cracking. Lightning strikes significantly degrade the uniaxial compressive performance of CFRP laminates, with induced delamination serving as the primary degradation mechanism.

**Figure 6:**
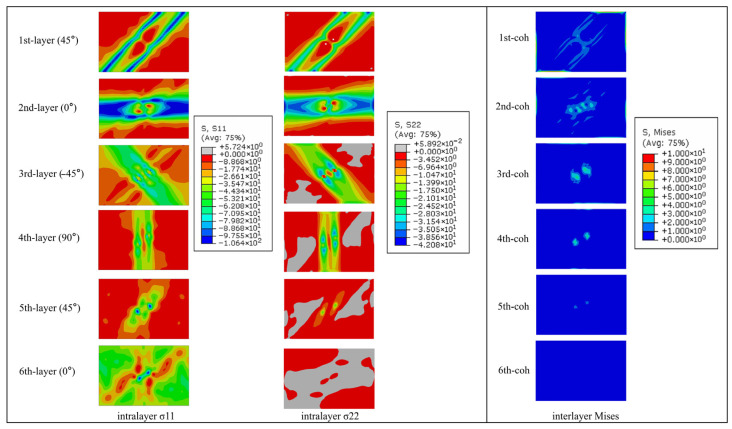
Contour of thermal stress distribution of laminate.

Figure 6 depicts the thermal stress distribution within the laminate during a lightning strike. Under four-edge clamped boundary conditions, in-plane deformation is severely constrained. This confinement induces out-of-plane thermal expansion at the laminate center, manifested as protrusion consistent with experimental warpage observations. Central regions experience reduced mechanical constraints in all three directions, resulting in lower triaxial thermal stresses. Predominantly compressive in-plane stresses align with fiber orientation trajectories. There is a part of the low-thermal-stress zone in the center of the first CFRP layer, and the damage in this part of the unit is serious, the stiffness is seriously reduced, and the thermal expansion has caused serious mechanical damage.

The ultimate compressive load obtained directly from the uniaxial compression simulation after the lightning strike without heat dissipation is 196.21 kN, while the load obtained after cooling to room temperature is 183.06 kN. Heat dissipation, involving thermal ablation and expansion, increases the delamination area and reduces the ultimate compressive load.

**Figure 7:**
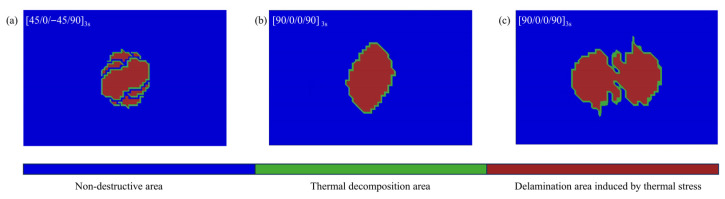
Superimposed contour of interlaminar damage in the laminate before compression: (**a**) [45/0/−45/90]_3s_ layup; (**b**) [90/0]_6s_ layup; (**c**) [90/0/0/90]_3s_ layup.

To evaluate the impact of different layups on thermal delamination damage, simulations were conducted to replicate the uniaxial compression process after lightning strikes on [45/0/−45/90]_3s_, [90/0]_6s_, and [90/0/0/90]_3s_ laminates under D-wave 40 kA lightning strike conditions. The lightning strike barely affects compressive stiffness. For the [45/0/−45/90]_3s_ layup, the compressive stiffness is 169.820 MPa, with an undamaged ultimate load of 257.50 kN and a post-lightning load of 183.06 kN, a reduction of 28.908%. For the [90/0]_6s_ layup, the stiffness is 200.583 MPa, with an undamaged ultimate load of 216.95 kN and a post-lightning load of 143.87 kN, a reduction of 33.685%. For the [90/0/0/90]_3s_ layup, the stiffness is 208.263 MPa, with an undamaged ultimate load of 260.131 kN and a post-lightning load of 139.651 kN, a reduction of 46.315%. A comparative analysis of the reduction in ultimate compressive load before and after lightning strikes across three different layup configurations reveals the following: the [45/0/−45/90]_3s_ layup exhibits the smallest reduction, the [90/0]_6s_ layup shows a moderate reduction, and the [90/0/0/90]_3s_ layup experiences the largest reduction. When compared to the [45/0/−45/90]_3s_ layup, the [90/0]_6s_ layup attains a lower peak temperature. However, it features a larger area of thermal decomposition in the first adhesive layer and a more extensive thermally induced delamination area, as shown in Figure 7. The reason is that the fibers in the first CFRP layer of the [90/0]_6s_ layup run along the short-edge direction. This orientation facilitates the rapid conduction of current outward, thereby decreasing joule heat generation and heat accumulation. As a result, ablation damage is predominantly localized near the surface. The [90/0/0/90]_3s_ layup shifts the symmetrical center of the [90/0]_6s_ layup forward. The third layer in this configuration more readily directs heat toward the 0° direction. Meanwhile, joule heat generation is more concentrated on the upper surface of the laminate. Since thermal expansion predominantly occurs along the fiber orientation, the first and second layers in the [90/0/0/90]_3s_ layup undergo significant misalignment during expansion. This misalignment leads to a larger thermally induced delamination area compared to the [90/0]_6s_ layup, resulting in more severe damage and consequently lowering the residual ultimate compressive load.

As shown in Table 4, ultimate compressive loads were calculated for the [45/0/−45/90]_3s_ laminate under variable D-waveform current amplitudes to quantitatively analyze current magnitude effects on thermally driven delamination damage. With the increase in current amplitude, the degradation degree of compressive performance of the laminate is significantly enhanced, and the ultimate load is 183.06 kN under 40 kA peak load, 147.22 kN under 60 kA peak load, and 115.61 kN under 80 kA peak load.

The C-waveform lightning current exhibits longer duration and lower amplitude than D-waveform strikes, resulting in reduced Joule heating but more pronounced thermal flux effects. This necessitates dual inputs of thermal flux and electrical current with convective cooling boundary conditions. Experimentally, load–displacement curves reveal more severe ablation damage under C-waveform exposure. Incorporating arc heating into the simulation increased the predicted ablation area by about 36 mm^2^ and thermal delamination area by about 10 mm^2^ compared to models excluding this mechanism, while reducing the post-strike ultimate compressive load by approximately 58.021 kN.

## 4. Conclusions

In this paper, a thermo-electro-mechanical coupling model considering cohesive effects is established, and the influences of delamination caused by thermal expansion on both the lightning strike process and the residual compressive strength of CFRP laminates are studied. The following conclusions can be obtained from the numerical results:

(1) The delamination pattern in each layer is primarily governed by the ply configuration of the overlying laminate. Additionally, a reduction in in-plane resistivity at the upper surface induces partial delamination in the non-pyrolyzed regions of the first cohesive layer.
(2) Incorporating delamination patterns into the model leads to a reduction in the intralaminar thermal damage area. This mitigation effect is attributed to energy absorption via interfacial delamination during lightning strikes, constituting 18.92% of the total energy dissipation. Critically, thermal expansion-induced delamination is not a valid contributor to this energy dissipation mechanism, in contrast to shockwave-driven delamination. Compared to the results from the thermo-electric coupling model, the predicted damage area due to lightning strikes aligns more closely with the C-scan image results.
(3) After being struck by a 40 kA D-waveform current, the ultimate compressive load of the CFRP laminate (183.06 kN, a 13.34% reduction from the non-cohesive model) obtained from the present model shows good agreement with the experimental result (158.93 kN). Specifically, the compressive strength degradation caused by lightning strikes is 36.72%, 28.91%, and 21.12%, corresponding to the experimental result and the simulation results with and without CZM, respectively. Through observation and comparison of damage contours under different loading, the presence of delamination indeed accelerates the propagation of intralaminar damage during compression, leading to lower strength.
(4) Delamination orientation in each layer is predominantly governed by the ply configuration of overlying laminate; reduced in-plane resistivity at the upper surface induces partial delamination in non-pyrolyzed regions of the first cohesive layer. Premature symmetry formation exacerbates delamination severity.

## Figures and Tables

**Table 1:** Temperature-dependent thermo-electric parameters of CFRP [34].

Temperature/K	Density/ (kg/m^3^)	Specific Heat/ (J/(kg·K))	Thermal Conductivity/(W/(m·K))			Electrical Conductivity/ (Ω·m)^−1^		
			*λ*_1_	*λ*_2_	*λ*_3_	*σ*_1_	*σ*_2_	*σ*_3_
298	1.52 × 10^3^	1065	8	0.67	0.67	0.03597	1.15 × 10^−6^	3.88 × 10^−9^
616	1.52 × 10^3^	2100	26.08	0.18	0.18	0.03597	1.15 × 10^−6^	3.88 × 10^−9^
773	1.1 × 10^3^	2100	1.736	1.736	1.736	0.03597	0.002	0.002
783	1.1 × 10^3^	1700	1.736	1.736	1.736	0.03597	0.002	0.002
1270	1.1 × 10^3^	1900	1.736	1.736	1.736	0.03597	0.002	0.002
3273	1.1 × 10^3^	2059	1.736	1.736	1.736	0.03597	0.002	0.002
3589	1.1 × 10^3^	2059	1.050	1.015	1.015	0.002	0.002	0.25
>3589	1.1 × 10^3^	5875	10^−5^	10^−5^	10^−5^	10^5^	10^5^	10^5^

**Table 2:** M21C mechanical properties (25 °C) [36].

Young’s modulus (GPa)	*E*_1_	146
	*E*_2_ = *E*_3_	9
Shear modulus (GPa)	*G*_12_ = *G*_13_	4.36
	*G*_23_	3
Poisson’s ratio	*ν*_12_ = *ν*_13_	0.3
	*ν*_23_	0.45
Thermal expansion coefficient (K^−1^)	*α*_1_	2.7 × 10^−6^
	*α*_2_ = *α*_3_	1.5 × 10^−5^
Penalty stiffness (N/mm^−3^)	7.73 × 10^9^	

**Table 3:** Traction and fracture toughness [39,40].

Mode I Interlaminar Maximum Traction $\sigma_{\max}$ (MPa)	Modes II, III Interlaminar Maximum Traction *τ_max_* (MPa)	Mode I Interlaminar Fracture Toughness $G_{\mathrm{IC}}$ (J/m^2^)	Modes II, III Interlaminar Fracture Toughness $G_{\mathrm{IIC}},\;G_{\mathrm{IIIC}}$ (J/m^2^)	Pyrolysis Degree (%)
65	100	435	1855	0
10^−4^	10^−5^	10^−6^	10^−7^	10
10^−4^	10^−5^	10^−6^	10^−7^	100

**Table 4:** Energy injected into laminate and post-lightning-strike compressive mechanical properties subjected to different current peaks of D-wave lightning strike.

Current Peak	Total Energy	Ultimate Compressive Strength
40 kA	2193.10 J	0.408 GPa
60 kA	3445.35 J	0.328 GPa
80 kA	4829.91 J	0.258 GPa

## Data Availability

The original contributions presented in this study are included in the article. Further inquiries can be directed to the corresponding authors.

## Abbreviations

The following abbreviations are used in this manuscript:

CALS	compression after lightning strike
CBLS	compression before lightning strike
CFRP	carbon fiber-reinforced polymer
CDM	continuum damage mechanics
CZM	cohesive zone modeling
CTEs	coefficients of thermal expansion
DTS	delamination induced by thermal stress
SWO	lightning strike-induced shockwave overpressure
W-CHSPs	woven composite honeycomb sandwich panels
